# Tracking individual broilers on video in terms of time and distance

**DOI:** 10.1016/j.psj.2023.103185

**Published:** 2023-10-11

**Authors:** J.E. Doornweerd, R.F. Veerkamp, B. de Klerk, M. van der Sluis, A.C. Bouwman, E.D. Ellen, G. Kootstra

**Affiliations:** ⁎ Animal Breeding and Genomics, Wageningen University & Research, 6700 AH Wageningen, the Netherlands; † Research & Development, Cobb Europe BV, 5831 GH Boxmeer, the Netherlands; ‡ Farm Technology, Wageningen University & Research, 6700 AA Wageningen, the Netherlands

**Keywords:** phenotype, computer vision, sensor, locomotion, deep learning

## Abstract

Tracking group-housed individual broilers using video can provide valuable information on their health, welfare, and performance, allowing breeders to identify novel or indicator traits that aid genetic improvement. However, their similar appearances make tracking individual broilers in a group-housed setting challenging. This study aimed to analyze broiler tracking on video (number of ID-switches, tracking time and distance) and examined potential tracking errors (ID-losses – location, proximity, kinematics) in an experimental pen to enable broiler locomotion phenotyping. This comprehensive analysis provided insights into the potential and challenges of tracking group-housed broilers on video with regards to phenotyping broiler locomotion. Thirty-nine broilers, of which 35 noncolor marked, were housed in an experimental pen (1.80 × 2.61 m), and only data at 18 d of age were used. A YOLOv7-tiny model was trained (n = 140), validated (n = 30), and tested (n = 30) on 200 annotated frames to detect the broilers. On the test set, YOLOv7-tiny had a precision, recall, and average precision (@0.5 – Intersection over Union threshold) of 0.99. A multi-object tracker (SORT) was implemented and evaluated on ground-truth trajectories of thirteen white broilers based on 136 min of video data (1-min intervals). The number of ID-switches varied from 5 to 20 (mean: 9.92) per ground-truth trajectory, tracking times ranged from 1 (by definition) to 51 min (mean: 12.36), and tracking distances ranged from 0.01 to 17.07 meters (mean: 1.89) per tracklet. Tracking errors primarily occurred when broilers were occluded by the drinker, and relatively frequently when broilers were in close proximity (within 10 cm), with velocity and acceleration appearing to have a lesser impact on tracking errors. The study establishes a ‘baseline’ for future research and identified the potential and challenges of tracking group-housed individual broilers. The results highlighted the importance of addressing ID-switches, identified potential tracking algorithm improvements, and emphasized the need for an external animal identification system to enable objective, simultaneous and semi-continuous locomotion phenotyping of group-housed individual broilers.

## INTRODUCTION

Individual broiler phenotypes are a cornerstone of broiler breeding programs for the genetic improvement of traits. However, manually phenotyping individual broilers for traits related to health, welfare, and performance is often a difficult and time-consuming task. Sensors can automatically collect individual broiler phenotypes at high temporal and spatial resolution on a large scale, providing possible novel or indicator traits (i.e., proxies) for broiler health, welfare, and performance. High-throughput phenotyping with sensors presents an opportunity to improve the effectiveness of selection for traits related to broiler health, welfare, and performance in broiler breeding programs.

Phenotypes of individual broilers collected through sensors, be it wearable or remote, often relate to the activity of the animals. Different sensors record different aspects of physical activity: some measure changes in locations (ultra-wideband [**UWB**], passive radiofrequency identification [**RFID**], video tracking; Van der Sluis et al., 2019, 2020, Doornweerd et al., 2023) and others changes in acceleration (linear and rotational; accelerometers [Yang et al., 2021] and inertial measurement units [**IMUs**; Derakhshani et al., 2022]). Changes in location and accelerations inform on the locomotory activity of broilers. Broiler locomotory activity could be considered a key indicator trait, as it was found to be related to broiler health (Hart, 1988), welfare (Van Hertem et al., 2018a), and performance (Tickle et al., 2018; Van der Sluis, 2022).

Different sensors, each with different characteristics, could be used for the high-throughput phenotyping of broiler locomotory activity (i.e., movement). In movement ecology, there are 4 defining criteria for sensors: temporal resolution (sampling rate), tracking duration (applicability/battery), concurrency (simultaneous tracking), and cost-effectiveness (Nathan et al., 2022). Depending on the goal of phenotyping and the phenotyping environment, different sensor characteristics become important (Williams et al., 2020). These characteristics include spatial scale (coverage of the system in the environment), spatial resolution (precision and accuracy), invasiveness (interference with the animal's behavior), applicability (species and animal age), and identification of individual animals (Nathan et al., 2022). These same criteria and characteristics can be applied for the high-throughput phenotyping of broilers.

Phenotyping broiler locomotion in commercial environments with different group sizes, stocking densities, and in different environments brings different sensor requirements. Wearable sensors are preferably small, lightweight, and have a battery life that can last the desired recording duration to maximize sensor applicability and minimize interference with the animal. However, they might not be the most cost-effective, as every animal requires a separate sensor. Alternatively, remote sensors such as video cameras present a potentially cost-effective method for phenotyping broilers. A single camera can record multiple animals (and their environment) and offers noninvasive observations with high spatio-temporal resolution. Nevertheless, tracking multiple group-housed individual broilers on video simultaneously is a challenging task.

Two studies, conducted by Van der Sluis et al. (2020) and Doornweerd et al. (2023), compared the use of video-based tracking to tracking based on UWB and RFID-sensors to phenotype broiler locomotion of color-marked broilers. The two studies used data collected in the same experiment with 4 color-marked broilers to compare the total distance moved but differed in their comparison approach and data subset. Both UWB and RFID were found to be suitable for estimating differences in distance moved between individual broilers (Van der Sluis et al., 2020; Doornweerd et al., 2023). However, both studies concur that video-based tracking outperformed UWB and passive RFID by providing more accurate location and movement data. This observation aligns with similar findings in other species, such as those reported in Redfern et al. (2017) using rats and Catarinucci et al. (2014) using mice. The consistency is largely attributed to the inherent functionality of the RFID-sensors. The RFID-system is constrained to movement between antennas, whereas video-based tracking offers unrestricted monitoring (Redfern et al., 2017; Van der Sluis et al., 2020; Doornweerd et al., 2023). Furthermore, the RFID-system may occasionally yield spurious or simultaneous readings, which can impact the accuracy of the location and movement data (Catarinucci et al., 2014; Van der Sluis et al., 2020; Doornweerd et al., 2023).

Video-based tracking, also known as Multi-Object Tracking (**MOT**), is the task of detecting objects within video frames and associating those detections across consecutive frames. In MOT, two paradigms exist: detection-and-tracking and tracking-by-detection, with the latter being more widely adopted, driven by improvements in object detection methods (Li et al., 2021). The distinction between the two lies in whether the detection and tracking tasks are performed jointly [detection-and-tracking, e.g., FairMOT (Zhang et al., 2020)] or separately [tracking-by-detection, e.g., SORT (Bewley et al., 2016)]. The MOT task becomes particularly challenging due to occlusions, similar appearances among objects, motion blur due to fast movement, and environmental factors (lightning conditions and obstacles). For instance, Guzhva et al. (2018) tracked cows in the waiting area of the automatic milking stations during the night (few cows, artificial light) and during the day (crowded, sunlit) over multiple camera views. The average tracking duration was 1.96 min during the night and 5.41 min during the day. Similarly, Van der Zande et al. (2023) argued that the tracking duration of pigs housed in pens with different stocking densities (57.8 min for 1.86 m^2^ pig^−1^ vs. 22.3 min for 0.93 m^2^ pig^−1^) was mostly influenced by the stocking densities and the positioning of the pig within the pen. However, these studies show that video shows promise as a viable method to track livestock.

In the study by Doornweerd et al. (2023), video-based tracking was evaluated for 3 color-marked broilers. However, in practice, broilers are not color-marked. On the contrary, broilers are typically group-housed in large numbers, where individuals share similar appearances. The aim of this study was to measure how long noncolor marked broilers could be tracked on video in terms of time and distance in an experimental pen. Furthermore, tracking errors were investigated to examine where in the pen they happened and when they happened (proximity and kinematics). This study provides insight into the video-tracking of white broilers using a popular tracking-by-detection algorithm (SORT; Bewley et al., 2016), identifies bottlenecks, and proposes potential solutions, and implications for phenotyping broiler locomotion.

## MATERIAL AND METHODS

### Ethical Statement

Data were collected under control of Cobb Europe. Cobb Europe complies with Dutch legislation on animal welfare. The Animal Welfare Body of Wageningen Research confirmed that this study was not an animal experiment under the Dutch Law on Animal Experiments.

### Data Acquisition

#### Animals and Housing

The experimental data have previously been described in van der Sluis et al. (2020) and Doornweerd et al. (2023). Thirty-nine male broilers, of which 35 were noncolor marked, from 2 genetic crosses, were housed in one pen (1.80 × 2.61 m). In the present study, video data at 18 d of age were used. Standard lighting conditions were maintained, and feed and water were provided ad libitum through 4 feeders and 1 drinker (Van der Sluis et al., 2020).

#### Video

A 2D (RGB) camera (Zavio B6210 2MP camera - Zavio Inc., Hsinchu City, Taiwan) placed above the pen recorded the birds from 10:44 AM to 12:59 PM at 18 d of age. The approximately 2-h recording period was divided into 20 videos of approximately 7 min each. Video recordings were made in full-HD (1920 × 1080 px) at 25 frames per second (for an example frame, see Figure 2). Video was used for human annotation of ‘ground-truth’ to train the broiler detection algorithm, to evaluate the detection of broilers within frames, and to evaluate the tracking of broilers between frames.

#### Annotation

**Figure 1:**
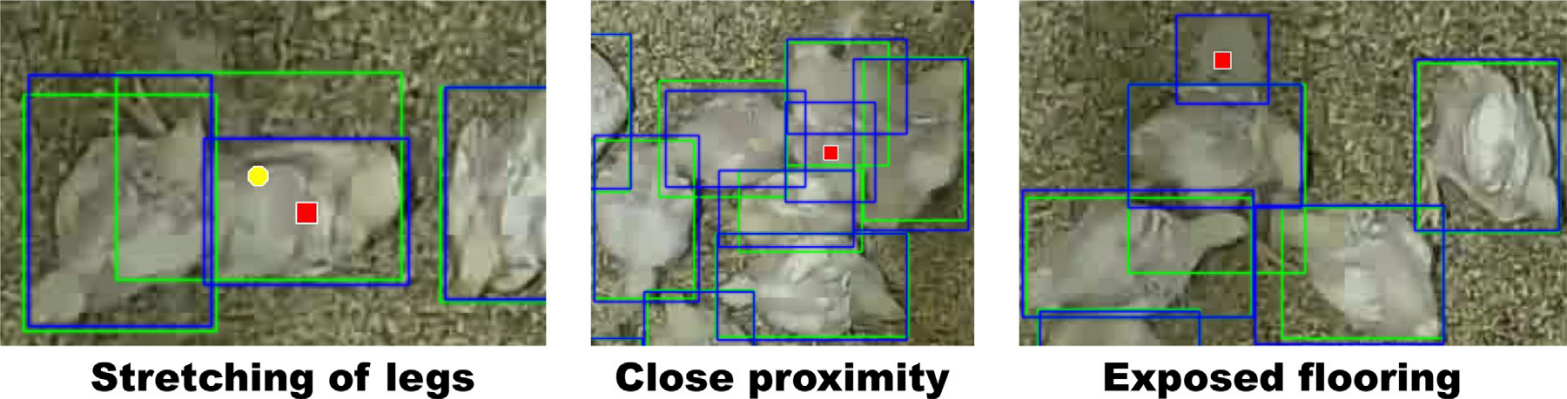
Ground-truth annotations (green) and YOLOv7’s detections (blue) in different situations with false positives (red square) and false negatives (yellow circle).

Video frames were annotated using the Computer Vision Annotation Tool (**CVAT**; Sekachev et al., 2019). All visible broilers (color-marked and noncolor marked) were annotated in each frame with a tight bounding box, including the entirety of the broiler, even if partially occluded. Bounding boxes were allowed to overlap. All annotations were annotated as one annotation class (broiler). To train and evaluate the broiler detection algorithm 200 randomly selected frames from the entire 2-h recording period were annotated by one human annotator (7,768 annotated broilers in total; examples in Figure 1).

For the evaluation of the tracker, ground-truth trajectories of 13 randomly selected noncolor marked broilers were annotated by 4 well-instructed researchers. The annotations were made at approximately 1-min intervals (every 1,500 frames, 136 frames in total) over the entire recording period. Each researcher annotated the ground-truth trajectory of 3 to 4 noncolor marked broilers across all 136 frames as a set of bounding boxes assigned with a unique ID which was consistent over time, i.e., the path of a specific broiler over time.

### Broiler Detection and Tracking

#### Broiler Detection

YOLOv7 (Wang et al., 2022) was used to detect the broilers on video within frames. In this study, the YOLOv7-tiny model pretrained on the Microsoft Common Objects in Context (**COCO**) dataset (Lin et al., 2014) was fine-tuned to detect broilers. The 200 annotated frames were randomly divided into a train set (n = 140 frames), a validation set (n = 30 frames), and a test set (n = 30 frames). The model was trained using default hyperparameters, apart from batch size which was lowered to 12 to fit GPU memory (Supplementary information). This trained YOLOv7-tiny model takes an input image and predicts bounding box coordinates, classes, and confidence scores for all the broilers in the video frames.

#### Broiler Tracking

Simple Online Realtime Tracking (SORT; Bewley et al., 2016) was used to track the detected broilers on video over consecutive frames. SORT associates the broiler detections between consecutive frames using Kalman Filters (Kalman, 1960) and the Hungarian algorithm (Kuhn, 1955). Hence, the detector's performance is crucial for the tracker's performance as the tracker relies on the accuracy and reliability of the broiler detections. In essence, SORT consists of 3 key steps for the frame-to-frame association of detections per time step t (prediction, data association, and an update step) further explained below.

In the first step, SORT predicts the location for each track in the next frame using a Kalman Filter. At each time step t, all object tracks $O_{t}=\;\left\{o_{1},\;\ldots ,\;o_{n}\right\}$ have a state estimate defined as $x={\left[x,y,\;s,r,\dot{x},\dot{y},\dot{s}\right]}^{T}$ and a corresponding state covariance matrix (P). In this state vector (x), (x,y) are the Cartesian coordinates of the object's center, s and rrepresent the scale (area) and aspect ratio (width / height) respectively, and $\dot{x},\dot{y}$ and $\dot{s}$ correspond to the respective velocities. Initially, the state is initialized based on the detections with velocities set to zero, and the covariance matrix set with high variances for the unobservable initial velocities. The predicted state [${\hat{x}}_{t|t-1}$, equation (1)] and state covariance matrix [$P_{t|t-1}$**,** equation (2)] are calculated as follows:

(1) $${\hat{x}}_{t|t-1}=Fx_{t-1|t-1}$$

(2) $$P_{t|t-1}=FP_{t-1|t-1}F^{T}+Q$$

Where F is the state transition matrix, which describes the dynamics of the system as a linear constant velocity model, and Q is the process covariance matrix which describes the uncertainty in the state transition.

In the second step, SORT associates the detected bounding boxes and predicted object tracks by similarity assessment. The association uses the Hungarian algorithm for optimal assignment between detected bounding boxes and predicted object tracks. Similarity is assessed for all detected bounding boxes and predicted object tracks as the intersection over union [**IOU**, based on bounding box positions and sizes, equation (3)] to form a cost-matrix as input for the Hungarian algorithm.

(3) $$\mathrm{IOU}\left(\hat{B},\;B\right)=\frac{\left|\hat{B}\cap B\right|}{\left|\hat{B}\cup B\right|}$$

Where $\hat{B}$ is the predicted bounding box, and B the detected or ground-truth bounding box depending on context.

A detected bounding box and a predicted object track are considered unmatched if the IOU between them is less than the minimum IOU-threshold (< 0.3, hyperparameter). An unmatched detected bounding box leads to the initialization of a new tentative track, as it could be an untracked broiler. This new tentative track must be associated with a minimum number of detections (3 matches in subsequent frames, minimum hits hyperparameter) before being assigned a tracking ID, as this avoids redundant tracking. Unmatched tracks are retained for a certain period (1 frame, maximum age hyperparameter) and are terminated if not matched in the subsequent frame(s). In this study, the hyperparameters were kept as default.

In the third step, SORT updates the state vectors $\left({\hat{x}}_{t|t-1}\right)$ and state covariance matrices $\left(P_{t|t-1}\right)$ of each track based on the associated detected bounding box $\left(z_{t}\right)$. The updated state vector $\left({\hat{x}}_{t|t}\right)$ is somewhere between the predicted state vector $\left({\hat{x}}_{t|t-1}\right)$ and the measurement $\left(z_{t}\right)$ weighted by the Kalman gain $\left(k_{t}\right)$. The measurement $\left(z_{t}\right)$ is defined as $z={\left[x,y,\;w,h,c\right]}^{T}$, where (x,y) are the Cartesian coordinates of the detected bounding box's center, w is the object width, h the object height, and c the detection confidence. The Kalman gain [$k_{t}$**,** equation (4)], updated state [${\hat{x}}_{t|t}$, equation (5)], and state covariance matrix [$P_{t|t}$**,** equation (6)] are calculated as follows:

(4) $$k_{t}=P_{t|t-1}H^{T}{\left[HP_{t|t-1}H^{T}+R\right]}^{-1}$$

(5) $${\hat{x}}_{t|t}=\;{\hat{x}}_{t|t-1}+\;k_{t}\left[z_{t}-H{\hat{x}}_{t|t-1}\right]$$

(6) $$P_{t|t}=\left[I-k_{t}H\right]P_{t|t-1}$$

Where H is the measurement matrix which maps to measurement space, R is the measurement covariance matrix which describes the uncertainty in the measurement, and I is the identity matrix.

### Evaluation

#### Evaluation Broiler Detection

The detection algorithm (YOLOv7) was evaluated based on how well it detected the broilers within frames. The number of true positives, false positives, and false negatives between ground-truth annotations and predictions formed the basis of the evaluation metrics. A prediction was a true positive, a false positive, or a false negative depending on the confidence score, classification, and the IOU [equation (3)] with the ground-truth annotation. A true positive refers to predictions that meet 3 criteria: a confidence score greater than or equal to the confidence threshold, a correct classification, and an IOU greater than or equal to the IOU-threshold. A false positive refers to predictions with a confidence score greater than or equal to the confidence threshold but with either the wrong classification or an IOU less than the IOU-threshold. A false negative refers to ground-truth annotations that did not match a prediction. The evaluation metrics were precision, recall, and average precision (**AP**). The AP was calculated for 2 IOU-thresholds ($\text{IOU}\geq \;\tau_{IOU}\;\in \left\{0.5,\;0.75\right\}$) resulting in AP@0.5 and AP@0.75. Precision and recall are reported for the confidence threshold that maximized F1 on the respective sets and an IOU-threshold of 0.5 (see also Padilla et al., 2021).

#### Evaluation Broiler Tracking

SORT was evaluated on its ability to track the 13 annotated white broilers between frames with the ground-truth trajectories. Note that all 39 animals were tracked, with annotated ground-truth trajectories for 13 broilers. To evaluate the tracking performance of SORT, ground-truth trajectories and tracker predictions needed to be matched. This approach was adopted from Luiten et al. (2021) and involved a one-to-one matching per frame, with multiple possible combinations of ground-truth trajectories and tracker predictions between frames. Optimal assignment between ground-truth trajectories and tracker predictions was determined using the Hungarian algorithm based on a global alignment score weighed by similarity (IOU) between ground-truth trajectories and tracker predictions. The global alignment score captures the alignment between all possible combinations of ground-truth trajectories and tracker predictions across the videos, with the aim of improving the consistency and accuracy of matching across multiple frames. The global alignment score is an overall Jaccard alignment score based on the count of potential matches between ground-truth trajectories and tracker predictions, the number of ground-truth trajectories, and tracker predictions across all evaluation frames. Per evaluation frame, the global alignment score is weighed by similarity to form a cost matrix as input for the Hungarian algorithm. Ground-truth tracks and tracker predictions required an IOU of at least 0.5 to be matched (see Luiten et al. (2021)). This process resulted in the matches between predicted object tracks using YOLO and SORT and the ground-truth trajectories.

In this study, evaluation metrics included the number of ID-switches per ground-truth track (i.e., per individual), tracking time in minutes per tracklet, and tracking distance in meters per tracklet. ID-switches were defined as changes in the ID associated with a ground-truth trajectory, while a tracklet referred to a track where a specific broiler had a consistently associated ID in sequential evaluation frames. Hence, a switch in the associated ID resulted in a new tracklet for the ground-truth trajectory of a specific broiler. ID-switches could be caused by the loss of the existing ID (a new ID is assigned), as well as swaps of IDs between tracks (give to, receive from, and exchange with another broiler). Defining ID-switches as a change in ID and not a swap (exchange) of IDs makes the metric stricter and easier to implement (i.e., a higher number of reported ID-switches; Li. Y. et al., 2009). The tracking distance per tracklet was converted from pixels to meters with a conversion factor derived from the centerline of the pen with a known length.

ID-losses of SORT were evaluated for all broilers (n = 39). ID-losses were defined as track IDs that were found in the previous frame but not in the current frame. The same track ID can be lost and re-appear multiple times due to the maximum age hyperparameter of SORT. In such cases, these instances were still considered an ID-loss. Four consecutive frames affected by faltering light were ignored, as it affected broiler detection.

To evaluate the circumstances under which ID-losses occurred, the last known location and timing of the lost ID were examined using the proximity to other broilers (distance to the nearest broiler detection; number of broilers) and their kinematics (velocity and acceleration), and compared these circumstances to situations where no ID-loss occurred. Proximity (the distance to the nearest broiler detection) was calculated as the Euclidean distance between the center point coordinates of the ID-losses’ last location and all other detections’ bounding boxes. The proximity to the nearest broiler for tracks where no ID-loss occurred did not factor in the distance to the lost ID. This omission was deliberate to ensure that only distances that did not potentially lead to an ID-loss were considered. Proximity was subdivided into 2 different proximity classes: proximity and close proximity. Broilers were considered in proximity if the distance between the broilers was less than or equal to 29 cm (≤ 128 px) and were in close proximity if the distance between the broilers was within the mean radius of a broiler (≤ 59 px, 13.5 cm), calculated from the ground-truth annotations. Kinematics (average velocity and acceleration) were calculated on the X and Y axis, with timesteps of 1 second, and reported as magnitudes [equation (7) and (8)]. Velocity and acceleration magnitudes were reported for the frame before the ID-loss had occurred for every broiler track.

(7) $$\left|\bar{V}\right|=\sqrt{{\left(\frac{x_{t}-x_{t-1}}{1}\right)}^{2}+{\left(\frac{y_{t}-y_{t-1}}{1}\right)}^{2}}$$

Where $|\bar{V}|$ represents the magnitude of velocity, and Δx and Δy denote the displacement in the x and y directions, respectively, over the change in time (Δt).

(8) $$\left|\bar{A}\right|=\sqrt{{\left(\frac{\Delta \mathrm{Vx}}{\Delta t}\right)}^{2}+{\left(\frac{\Delta \mathrm{Vy}}{\Delta t}\right)}^{2}}$$

Where $|\bar{A}|$ represents the magnitude of acceleration, and ΔVx and ΔVy denote the changes in velocity in the x and y directions, respectively, over the change in time (Δt).

## RESULTS

### Broiler Detection Performance

Broiler detection performance is important for broiler tracking performance when using tracking-by-detection algorithms. Therefore, the model was evaluated on how well it detected the broilers within frames of the annotated dataset. The YOLOv7-tiny model performed well and could detect group-housed broilers accurately (Table 1; Figure 1). It achieved a precision, recall and AP@0.5 of 0.99 and an AP@0.75 of 0.98, indicating that increasing the IOU-threshold had little impact on the model's precision. Although there were some false positives and false negatives, the model successfully identified nearly all the broilers in the test set. False positives in the test set occurred due to stretching of legs (n = 2), exposed flooring (n = 1), and in situations with broilers in close proximity (n = 1; Figure 1). A false negative occurred in the test set where a broiler was annotated with a stretched leg, but the stretched leg was not detected as belonging to that specific broiler (n = 1, Figure 1).

**Table 1:** Detection performance of YOLOv7-tiny on train, validation, and test set. Average precision (AP) shown for an intersection over union (IOU) of 0.5 and 0.75. Precision and recall for an IOU-threshold of 0.5 and confidence threshold (^a, b, c^) that maximized F1. Values were calculated for each dataset and for each image within its respective dataset, expressed as [mean (SD)].

Metric	Train^a^	Validation^b^	Test^c^
Precision	0.99 [1 (<0.01)]	0.99 [0.99 (<0.01)]	0.99 [0.99 (<0.01)]
Recall	1.00 [1 (<0.01)]	0.99 [0.99 (<0.01)]	0.99 [0.99 (<0.01]
AP@0.5	0.99 [0.99 (<0.01)]	0.99 [0.99 (<0.01)]	0.99 [0.99 (<0.01]
AP@0.75	0.99 [0.99 (0.010)]	0.98 [0.98 (0.034)]	0.98 [0.98 (0.020)]

a Confidence threshold: 0.654.

b Confidence threshold: 0.639.

c Confidence threshold: 0.821.

### Broiler Tracking Performance

**Figure 2:**
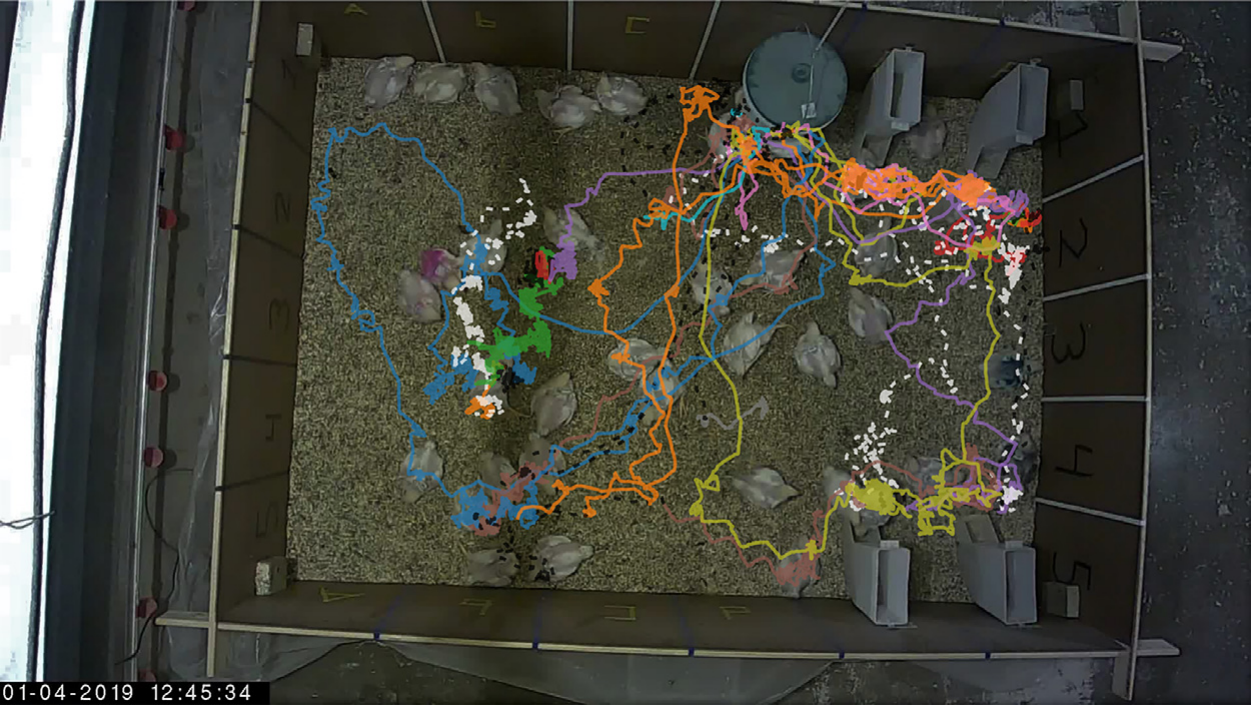
Tracklets of the broiler with the most ID-switches. Locations shown are over the entire duration of the recording period (135 min). Each tracklet is represented with a different color. Dashed lines indicate part of the tracklet that existed prior to ground-truth assignment (white) or that continued after the last ground-truth assignment (switch took place in the interval between ground-truth frames or ID-switch; black). The plot is projected over a darkened frame.

The ability to track broilers over time accurately and reliably is key to collecting reproducible phenotypic information linked to a specific individual. The evaluation of broiler tracking performance involved the comparison of the trajectories of SORT with the ground-truth trajectories of 13 broilers. A matching step was performed to align SORT's predicted trajectories to the corresponding ground-truth trajectory of the individual broilers. Afterward, it is possible to recover the assumed complete trajectory of an individual broiler over the entire recording period based on all aligned SORT trajectories (Figure 2). An individual broiler's trajectory consists of multiple tracklets of varying lengths, and a single tracklet may include segments of the trajectories of several other broilers due to the ID-switches.

**Figure 3:**
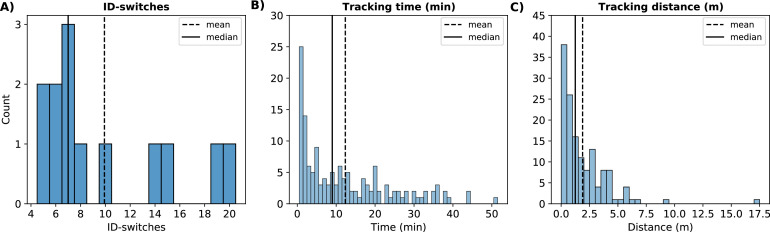
Tracking performance of SORT in ID-switches (#; A) per broiler ground-truth trajectory, tracking time (min; B) and tracking distance (m; C) per tracklet.

**Figure 4:**
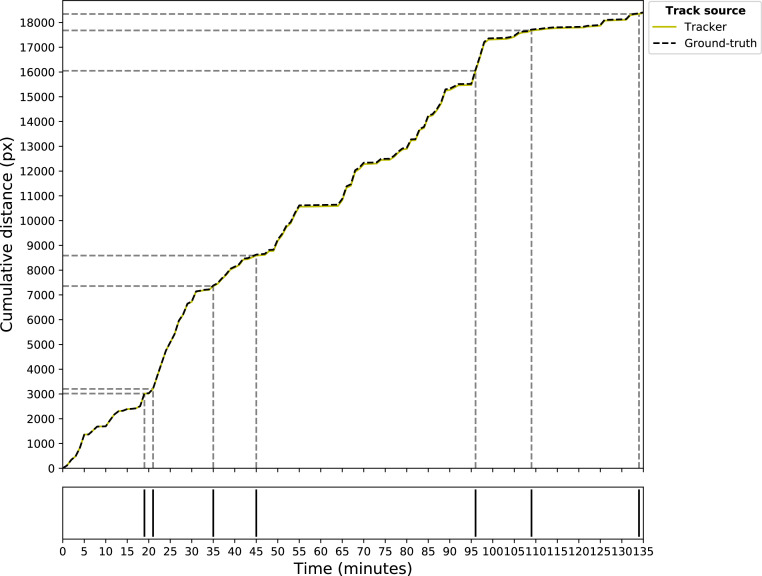
Broiler tracking plot of the broiler with the longest tracking duration and tracking distance. The plot shows ID-switches (gray dashed lines, bottom plot), tracking duration (in min on the x-axis) and tracking distance (in px on the y-axis) per tracklet. The ground-truth trajectory is represented by a black dashed line, and the associated SORT tracks depicted by a yellow solid line. This particular broiler had 7 ID-switches, an average tracking time of 16.88 min, and average tracking distance of 5.26 m per tracklet.

**Figure 5:**
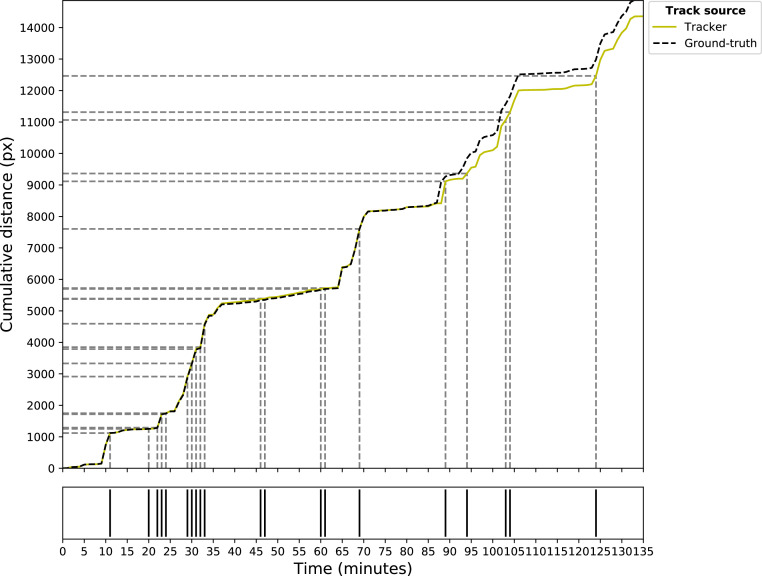
Broiler tracking plot of the broiler with the most ID-switches. The plot shows ID-switches (gray dashed lines, bottom plot), tracking duration (in min on the x-axis) and tracking distance (in px on the y-axis) per tracklet. The ground-truth trajectory is represented by a black dashed line, and the associated SORT tracks depicted by a yellow solid line. This broiler had 20 ID-switches, an average tracking time of 6.43 min and average tracking distance of 1.56 m per tracklet.

The number of tracklets varied for each individual ground-truth trajectory and was determined by quantifying the number of ID-switches per ground-truth trajectory (min: 5, mean: 9.92, max: 20; Figure 3A). Generally, a higher number of ID-switches per ground-truth trajectory were associated with more tracklets of shorter durations and distances. However, the lengths of the tracklets could still vary within an individual broiler's ground-truth trajectory. On average, tracklets had a duration of 12.36 min, with the longest tracklet lasting 51 min and the shortest 1 min (by definition; Figure 3B). The longest tracking distance was 17.07 m, whereas the average tracking distance per tracklet was 1.89 m. The shortest tracking distance observed was 0.01 m (Figure 3C). The broiler with the longest tracking distance (17.07 m) also had the longest tracking duration (51 min; Figures 3 and 4). The broiler with the most ID-switches (20) had 2 tracklets with a tracking time of 20 min with tracking distances of 3.46 m and 2.64 m for those 2 tracklets (Figures 3 and 5).

The tracklets produced by SORT showed good alignment with the broiler ground-truth tracks (Figures 4 and 5). Any observed offset between the two resulted potentially from occlusion or small differences between the actual location and predicted locations, as the tracks remained parallel (Figure 5). Consequently, the total cumulative distance differed between the ground-truth trajectory and the assumed trajectory of an individual broiler.

### ID-Losses

To identify potential tracking errors and their potential underlying causes, the occurrence of ID-losses, the loss of track IDs in the current frame compared to the previous one, was evaluated. In this study, all 39 broilers were detected and tracked throughout the entire recording period, which spanned 135 min. This coverage allowed for a thorough investigation of ID-losses.

#### Location

**Figure 6:**
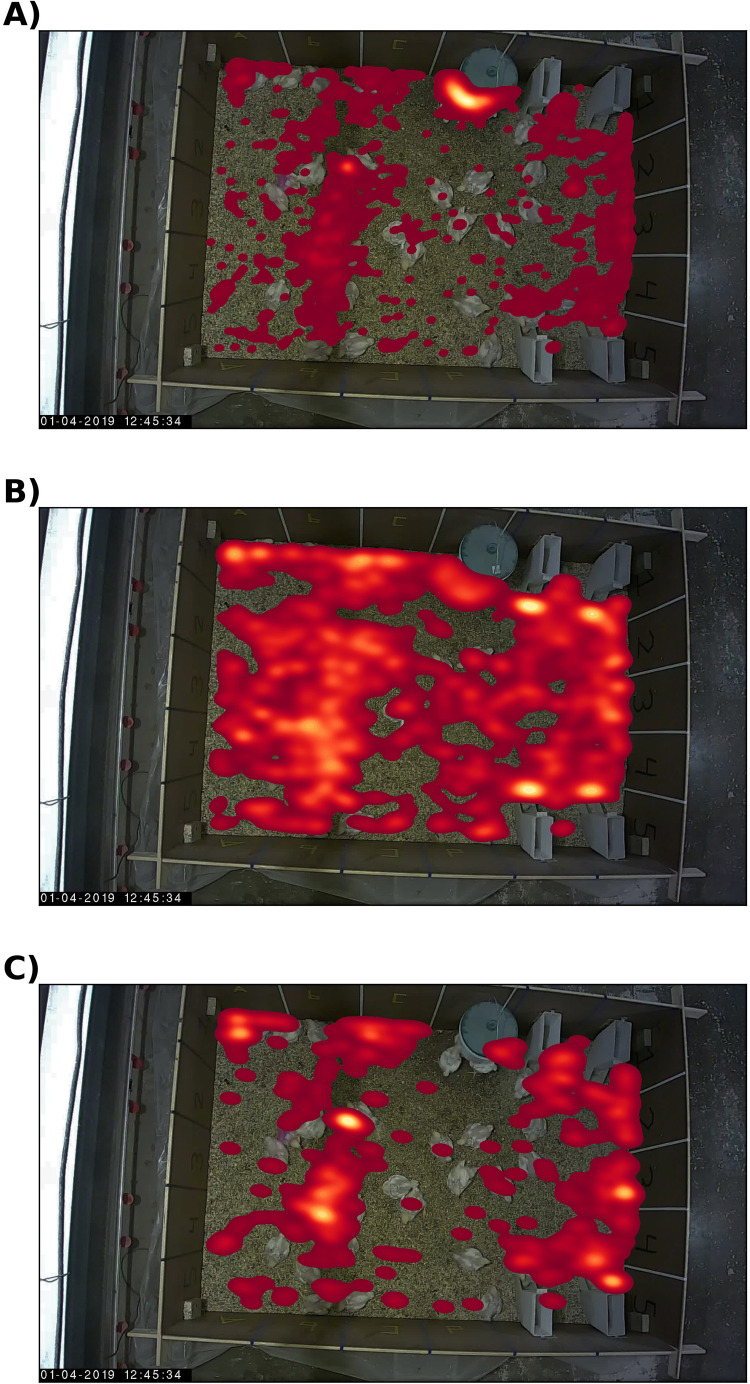
Heatmaps of the last locations of IDs before ID-loss (A), the location of all broiler detections over time (B, 1 FPS), and the last locations of IDs with more than 1 second existence and excluding ID-losses that occurred near the drinker (C). The animals’ bounding box center points were used to determine the locations. The plot is projected over a darkened frame.

A total of 1,952 ID-losses were observed during the entire duration of the recording period. These 1,952 ID-losses occurred throughout the pen, but hotspots could be seen around the B/C-column (marked on the wall of the pen) and the drinker (Figure 6A). The broilers spent most of their time within the B/C-column (shadow area), and therefore there were more opportunities for ID-losses (Figure 6B). Approximately 43% of the ID-losses occurred around the drinker, excluding these ID-losses resulted in a decrease in the total number of ID-losses to 1,114 (from 1,952 with the drinker, Figure 6C).

As shown earlier, the broiler detection model performed well. However, it was not flawless, as indicated by the presence of false positives and false negatives in the test set. False positives that existed for more than the minimum number of detections (hyperparameter of SORT) were assigned an identity which subsequently caused an ID-loss, i.e., a false track not associated with a broiler. In certain cases, an ID would be lost, re-appear, and then be lost again; each of these instances counted as an ID-loss. The number of unique IDs that were lost informed on the frequency of lost and re-appeared occurrences. For example, the number of lost unique IDs decreased from 1,669 (accountable for 1,952 ID-losses) to 927 (1,114 ID-losses) after excluding ID-losses at the drinker. Most (602 IDs) of the 927 IDs existed for less than one second in total. Considering only IDs that existed for one or more seconds in total (325 IDs), the total number of ID-losses decreased to 489 (from 1,114 ID-losses). In the following part, the proximity and kinematics before the occurrence of the ID-loss are explored to investigate if they were potential reasons for ID-losses.

#### Proximity

**Figure 7:**
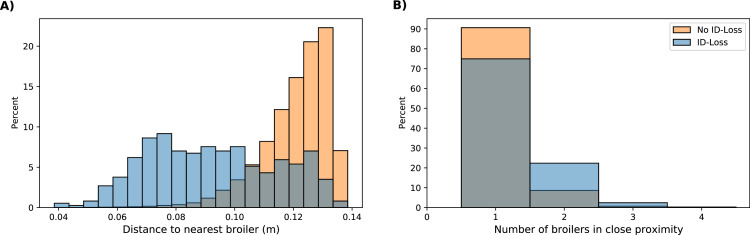
Proximity plot for both ID-loss detections (blue) and no ID-loss detections (orange). The plot displays 2 metrics: the Euclidean distance to the nearest broiler (A) and the number of broilers in close proximity (≤ 59 px, 13.5 cm). The y-axis represents the percentage of the total for each metric for ID-loss detections and no ID-loss detections.

Out of the 489 ID-losses with IDs that existed for one or more seconds, 471 occurred in proximity (≤ 128 px, 29 cm) to other broilers, and 371 of those instances occurred in close proximity (≤ 59 px, 13.5 cm) to other broilers. The proximity to the nearest broiler (Euclidean distance), and the number of broilers in close proximity are shown in Figure 7. In general, and relative to the situations where no ID-loss occurred (total of 1,535,999), ID-losses in close proximity to other broilers (total of 371) were relatively more frequent when broilers were closer to each other. However, it is important to note that ID-losses occurred at all distances that were within the range of close proximity (≤ 59 px, 13.5 cm, Figure 7A). For ID-loss detections, the average distance to the nearest broiler was 9.24 cm; for no ID-loss detections it was 12.08 cm. However, the minimum and maximum distance of the ID-loss and no ID-loss detections were similar [3.92 and 3.85 cm; 13.48 and 13.50 cm (by definition), respectively]. Considering the number of broilers in close proximity at the time of an ID-loss, the majority of ID-loss and no ID-loss detections occurred in close proximity (≤ 59 px, 13.5 cm) to a single broiler rather than multiple broilers (Figure 7B). Proximity to other broilers does appear to play a role in the occurrence of ID-losses, however, it is important to highlight that, more often than not, no ID-losses occur in close proximity.

#### Kinematics

**Figure 8:**
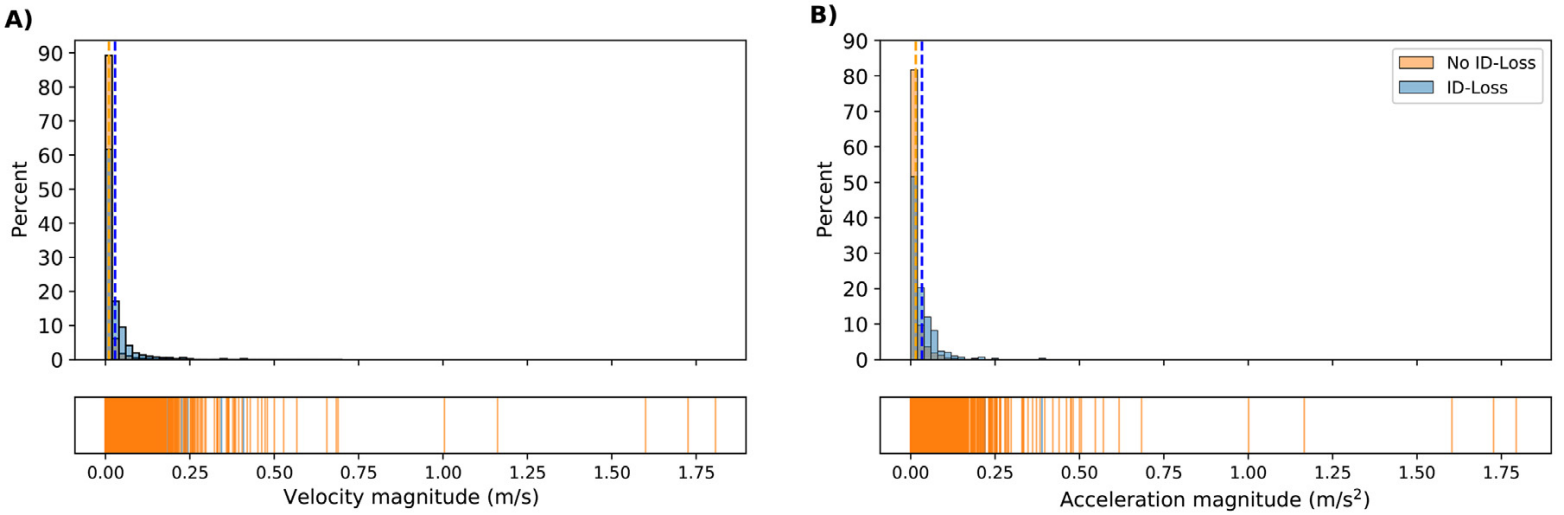
Kinematic plots of velocity (A) and acceleration (B) magnitudes for ID-loss detections (blue) and no ID-loss detections (orange) at the time of an ID-loss. The vertical dashed lines indicate the means. The y-axis represents the percentage of the total for each metric for ID-loss detections and no ID-loss detections.

Velocity and acceleration magnitudes were compared to see whether rapid movements were a reason for ID-losses (Figure 8). Velocity and acceleration magnitudes were comparable between ID-loss and no ID-loss detections. The majority of the ID-loss and no ID-loss detections, occurring in the same frame, had velocity and acceleration magnitudes less than 0.025 m/s and 0.025 m/s^2^ at the time of an ID-loss. On average, ID-loss detections had higher velocity and acceleration magnitudes than no ID-loss detections (0.028 m/s and 0.033 m/s^2^ vs. 0.010 m/s and 0.015 m/s^2^). However, no ID-loss detections had higher maximum velocity and acceleration magnitudes than ID-loss detections (1.81 m/s and 1.79 m/s^2^ vs. 0.41 m/s and 0.39 m/s^2^, respectively). In relative terms, ID-loss detections tended to have higher velocity and acceleration magnitudes, but kinematics do not seem to play a large role in causing ID-losses.

## DISCUSSION

This study analyzed the tracking of group-housed broilers on video in terms of time and distance in an experimental pen. ID-losses were examined to elucidate where and when they happened in terms of proximity and kinematics. A major strength of the study are the insights it provided into the potential and challenges of tracking group-housed broilers on video in the view of phenotyping broiler locomotion.

### Broiler Detection Performance

In tracking-by-detection, broiler detection accuracy is important, as the tracking algorithm relies on the quality of the broiler detections. In this study, detection accuracy metrics (recall, precision and AP@0.5 & AP@0.75) were 0.98 and above. However, these metrics are not directly comparable with other studies due to the substantial variation in recording conditions. These recording conditions encompass diverse factors such as group size, stocking density, broiler age, lighting and background. The detection performance is context-dependent and hence influenced by these factors, making a direct comparison between studies difficult. Nevertheless, the comparison can still offer insights into the challenges associated with broiler detection.

Various studies have used different versions of YOLO to detect layers or broilers in different environments. Wang et al. (2019) used YOLOv3 to detect layers and roosters within a cage-system (0.072 m^2^ bird^−1^, 330 d of age) and reported a mean AP@0.5 of 0.92 on their validation set (939 frames). Jaihuni et al. (2023) used YOLOv5 to detect broilers at different stocking densities (0.125 m^2^ bird^−1^ and 0.094 m^2^ bird^−1^) and different ages (4 to 47 d of age). They reported an AP of 0.98, assumed to be reported on their test set with an IOU-threshold of 0.5. In contrast, Chen et al. (2023) used YOLOv7-tiny to detect broilers (0.072 m^2^ bird^−1^, from 28 to 80 d of age), and they reported an AP@0.5 of 0.94 on their test set. In comparison to this study, the reported AP@0.5’s were lower. However, in this study stocking density was lower (0.12 m^2^ bird^−1^) and only data at 18 d of age were used. Nevertheless, similar reasons for false positives and false negatives were found, and were related to occlusions and proximity (Wang et al., 2019; Chen et al., 2023). These studies demonstrate the adaptability of YOLO-based models for poultry detection across different environments, as well as the shared challenges associated with poultry detection in different environments.

### Broiler Tracking Performance

Broiler tracking performance was evaluated with 3 metrics: ID-switches, tracking distance per tracklet, and tracking time per tracklet. It is important to consider all 3 metrics in unison to assess the tracking performance accurately as the metrics are interconnected. Shorter tracklet durations and distances were observed with an increase in ID-switches per ground-truth track. ID-switches include ID-swaps, which occur when IDs are transferred, received, or exchanged between broilers. Hence, an individual broiler's trajectory may consist of several tracklets with different IDs, and those individual tracklets may contain sections of the trajectories of other broilers due to ID-swaps. In an ideal situation, a ground-truth trajectory would have none or only a few ID-switches, along with long tracking distances and long tracking times per tracklet.

In this study, the evaluation frames had a 1-min interval. An ID-switch could happen at any point within the 1-min interval. The reported tracking durations and tracking distances are likely less accurate for tracklets with shorter durations. Particularly for tracklets with a longer duration, tracking distances per tracklet may be underestimated as movement is missed because of the interval and the accumulation of errors. Longer tracking durations did not necessarily equate to longer tracking distances, i.e., tracking inactive broilers is easier. Hence, shorter track durations (e.g. 10 min) in which the animal is active may yield more phenotypic information compared to longer track durations (e.g. 20 min) where the animal is inactive.

The minimal required tracking distance and tracking duration to phenotype broiler locomotion are unclear. Li et al. (2023b) conducted a study on phenotyping broiler locomotion from a top-view camera, utilizing a 1.5 m walkway and encouraging the birds to walk. Their videos (12 in total – 1 per bird; 4 per gait score) were between 0.5 and 3.5 min long. They found a significant difference in the forward acceleration of broilers with different gait scores, with better-walking birds having faster accelerations (Li et al., 2023b). Jaihuni et al. (2023) used a combination of YOLOv5 and a customized DeepSORT and reported tracking durations of at least 3 min on average. However, they comment that the lower tracking durations (3 min. compared to 12.36 min. in this study) may be appropriate to assess broiler locomotion. This suggests that shorter tracklets, in terms of distance and time, with activity may be appropriate to assess broiler locomotion. However, multiple tracklets from the same individual broiler may help improve the assessment of its locomotion, due to the repeated observations.

### ID-Losses

The investigation of ID-losses and their underlying causes revealed that the primary factors contributing to ID-losses were associated with location and proximity. A majority of ID-losses (approximately 56%, excluding drinker-associated ID-losses) could be attributed to false positives that persisted for a minimum of 3 frames, received an ID, and existed for less than one second in total. False positives in the test set (n = 4) showed that these occurred with broilers in close proximity, exposed flooring, and stretching legs. Watching some of the video footage revealed that false positives also occurred during preening and neck stretches and that false negatives occurred due to broiler-broiler occlusion and seemingly at random. False positives due to close proximity, exposed flooring, preening, and neck stretches could be resolved by improving the detection algorithm (more training data or different a algorithm) and adjusting the minimum hits hyperparameter of SORT required for track initialization. The latter is easiest to implement, as providing sufficient examples for the problematic situations might be difficult. Nonetheless, increasing the size of the dataset allows for a larger test set which gives a better assessment of false positive and false negative detections. However, given the current performance of the detection algorithm, the return of investment compared to tuning the minimum hits hyperparameter might be small. Increasing the minimum hits hyperparameter will shorten the tracklets a few frames but may indirectly filter false positive detections.

Although ID-losses occurred throughout the pen, a large proportion (42.93%) occurred near the drinker, and were likely lost due to occlusion, and not recovered due to unpredictable movement under the drinker and the maximum age hyperparameter of SORT for unmatched tracks (1 frame). By focusing only on ID-losses where the ID existed for more than one second and not near the drinker, it was found that these losses were relatively more common when broilers were in closer proximity. Velocities and accelerations could have played a role in ID-losses but did not appear to be the predominant cause of ID-losses. ID-losses did have higher velocity and acceleration magnitudes on average, however, most other IDs that were not lost at the time of an ID-loss had low velocity and acceleration magnitudes. A possible ID-loss scenario could be a broiler moving close to another broiler, nudging it, and causing an ID-loss, which could explain the proximity and kinematics findings. The proximity and nudge could affect both the detection and tracking algorithms. The detection algorithm may miss one of the broilers (false negative) due to broiler-broiler occlusion, which results in an ID-loss. However, assuming that the occluded broiler moves very little, the lost ID could be recovered by tuning the maximum age hyperparameter of SORT. Additionally, the sudden nudge could result in an ID-switch, as one broiler's bounding box suddenly shifts whereas the other's abruptly stops moving.

Based on the video footage, it can be speculated that sudden changes in movement, such as described above, or a sprint can affect the tracking and detection algorithms. In case of a sprint, the tracker could underestimate the movement, and tracker prediction and detections will be unable to match, i.e., no overlap. At the same time, fast movement results in a motion blur, obscuring the broiler, which leads to potentially missed detections or multiple detections for the fast-moving broiler. The tracker prediction error propagates, and a new ID is initialized on one of the potential multiple detections for the fast-moving broiler. This mismatch between tracker predictions and detections could be solved by improving the motion estimation (Cao et al., 2022) or improving the similarity assignment process of the tracking algorithm for the tracker predictions (Yang et al., 2022). Yang et al. (2022) proposed a cascaded buffered IOU (**C-BIoU**) to improve the similarity assignment process and replaced the Kalman Filter (Kalman, 1960) with a simpler motion estimation model. The cascaded buffered IOU allows for the matching of nonoverlapping detections and compensates for motion estimation errors. Similarity assignment with C-BIoU is cascaded with different buffers for matched and unmatched tracks to reduce the risk of mismatches. The simpler motion estimation model (average motion over recent frames) can quickly respond to unpredictable movement. Tuning hyperparameters of the current tracking algorithm or replacing it with C-BIoU could reduce ID-losses and improve tracker performance.

### Phenotyping Locomotion

Phenotyping individual broiler locomotion of group-housed broilers requires that tracklets are linked to a specific broiler. In practice, individual broiler trajectories from video will consist of a collection of tracklets linked to a specific broiler over a certain period of time. In some cases, these tracklets will include parts of the trajectories of several different broilers due to ID-swaps. The tracklets, or parts of them, will need to be assigned to the correct individual broiler in order to use these locomotion phenotypes for breeding. The assignment of tracklets to an individual broiler has two main complicating factors: ID-switches and the unique identification system.

ID-switches are a consequence of detection and tracking as per any tracking-by-detection method. One effective solution to reduce ID-switches is tuning the tracking algorithm's hyperparameters or optimizing the tracking algorithm. This hyperparameter tuning could be done empirically, with a sensitivity analysis, or using the domain knowledge obtained in this study. Nonetheless, ID-switches are probably not completely preventable, especially in situations with higher stocking densities such as those encountered in commercial environments. At higher stocking densities, broilers have a higher tendency to step on or over other broilers (Hall, 2001). This situation poses a challenge for the tracking and detection algorithms, potentially leading to a higher number of ID-switches, as observed in this study. In that case, shorter tracklets consisting of parts of the trajectory of one broiler would be preferred over longer tracklets consisting of parts of the trajectories of multiple broilers, i.e., fewer ID-swaps, but more ID-losses.

An animal identification system, independent of video, could be combined with video to phenotype individual broiler locomotion. Furthermore, the animal identification system could allow longer tracklets with potential ID-swaps to be dissected into different shorter tracklets belonging to different individual birds. Such approaches, for example using a combination of video tracking and a passive radio frequency identification (RFID) system have been used before in different livestock species and in different settings (Nakarmi et al., 2014; Guzhva et al., 2018; van Hertem et al., 2018b). However, in the poultry setting, there are some complications that require further research. This includes determining the placement and number of RFID-readers needed, as well as the development of the algorithm to link tracklets and parts of tracklets to specific individual broilers.

To fully assess the usefulness of the locomotion phenotypes as novel or indicator traits for breeding purposes, a detailed genetic study is essential. This genetic study would evaluate the repeatability, heritability, and genetic correlations of the locomotion phenotype to other traits. It would also provide insight into the optimal intensity of measuring the locomotion phenotype. The combination of video tracking and RFID will enable breeders to objectively, simultaneously, and (semi-)continuously phenotype the locomotion of numerous individual broilers in a group-housed setting.

## CONCLUSION

This study conducted a comprehensive analysis of tracking group-housed broilers on video to provide insight into the potential and challenges of phenotyping broiler locomotion from video. The analysis included the examination of broiler tracking performance, with a focus on time and distance metrics, as well as an examination of potential tracking errors to determine their cause and to propose potential solutions. In terms of broiler tracking performance, the number of ID-switches varied from 5 to 20 (mean: 9.92) per ground-truth trajectory, tracking distances ranged from 0.01 to 17.07 meters (mean: 1.89) and tracking times ranged from 1 (by definition) to 51 min (mean: 12.36). In practical terms, this signifies that an individual broiler's trajectory consists of multiple tracklets of varying lengths, and within a single tracklet, there may be segments from the trajectories of several broilers. Examination of the potential tracking errors (ID-losses) revealed that the majority were associated with the location in the pen (occluded by the drinker) and the proximity to other broilers (relative to no ID-losses, within 10 cm). Kinematics appeared to play a less predominant role in the occurrence of ID-losses. This comprehensive study provided insight into the potential and challenges of tracking group-housed individual broilers to phenotype broiler locomotion from video data. The analysis of broiler tracking performance and examination of potential tracking errors establishes a ‘baseline’ for expectations when tracking group-housed individual broilers. Future broiler locomotion phenotyping will not only require addressing ID-switches, either reduce them or favor ID-losses, and the optimization of the tracking algorithm, but also the use of an external animal identification system (e.g., passive radio frequency identification). An external animal identification system will help to assign tracklets, or parts of tracklets, to individual broilers for individual broiler locomotion phenotypes. The combination of the systems will enable breeders to objectively, simultaneously, and semi-continuously phenotype the locomotion of group-housed individual broilers.

## Appendix Supplementary materials

Image, application 1

## Footnotes

Supplementary material associated with this article can be found in the online version at doi:10.1016/j.psj.2023.103185.
