# Supplementary material for: Tracking individual broilers on video in terms of time and distance
Source: Poult Sci. 2023 Oct 11;103(1):103185. doi: 10.1016/j.psj.2023.103185 (PMC10663953; doi:10.1016/j.psj.2023.103185)
Supplement: Supplementary file 1 [file mmc1.docx]

**Supplementary Information**

**TRACKING INDIVIDUAL BROILERS**

**Tracking individual broilers on video in terms of time and distance**

**J.E. Doornweerd*^1^, R.F. Veerkamp*,** **B. de Klerk^†^, M. van der Sluis*, A.C. Bouwman*, E.D. Ellen*, and G. Kootstra^‡^**

^*^ Animal Breeding and Genomics, Wageningen University & Research, 6700 AH Wageningen, the Netherlands
^†^ Research & Development, Cobb Europe BV, 5831 GH Boxmeer, the Netherlands
^‡^ Farm Technology, Wageningen University & Research, 6700 AA Wageningen, the Netherlands
^1^ [janerik.doornweerd@wur.nl](mailto:janerik.doornweerd@wur.nl)
Present address: Wageningen Campus, Building 107, Droevendaalsesteeg 1, 6708 PB, Wageningen, the Netherlands.

**Hyperparameters**

lr0: 0.01 # initial learning rate (SGD=1E-2, Adam=1E-3)

lrf: 0.1 # final OneCycleLR learning rate (lr0 * lrf)

momentum: 0.937 # SGD momentum/Adam beta1

weight_decay: 0.0005 # optimizer weight decay 5e-4

warmup_epochs: 3.0 # warmup epochs (fractions ok)

warmup_momentum: 0.8 # warmup initial momentum

warmup_bias_lr: 0.1 # warmup initial bias lr

box: 0.05 # box loss gain

cls: 0.3 # cls loss gain

cls_pw: 1.0 # cls BCELoss positive_weight

obj: 0.7 # obj loss gain (scale with pixels)

obj_pw: 1.0 # obj BCELoss positive_weight

iou_t: 0.20 # IoU training threshold

anchor_t: 4.0 # anchor-multiple threshold

# anchors: 3 # anchors per output layer (0 to ignore)

fl_gamma: 0.0 # focal loss gamma (efficientDet default gamma=1.5)

hsv_h: 0.015 # image HSV-Hue augmentation (fraction)

hsv_s: 0.7 # image HSV-Saturation augmentation (fraction)

hsv_v: 0.4 # image HSV-Value augmentation (fraction)

degrees: 0.0 # image rotation (+/- deg)

translate: 0.2 # image translation (+/- fraction)

scale: 0.5 # image scale (+/- gain)

shear: 0.0 # image shear (+/- deg)

perspective: 0.0 # image perspective (+/- fraction), range 0-0.001

flipud: 0.0 # image flip up-down (probability)

fliplr: 0.5 # image flip left-right (probability)

mosaic: 1.0 # image mosaic (probability)

mixup: 0.0 # image mixup (probability)

copy_paste: 0.0 # image copy paste (probability)

paste_in: 0.0 # image copy paste (probability), use 0 for faster training

loss_ota: 1 # use ComputeLossOTA, use 0 for faster training
